# Perceptions of grandmothers and HIV-infected mothers on infant feeding practices in a rural South African district

**DOI:** 10.4102/hsag.v25i0.1372

**Published:** 2020-08-05

**Authors:** Motlatso G. Mlambo, Karl Peltzer

**Affiliations:** 1 Department of Institutional Research and Business Intelligence, Risk and Advisory Services, University of South Africa, Pretoria, South Africa; 2 Department of Family Medicine and Primary Care, Faculty of Health Sciences, University of the Witwatersrand, Johannesburg, South Africa; 3 Department of Research Administration and Development, University of Limpopo, Turfloop, South Africa

**Keywords:** PMTCT, infant feeding, mixed feeding, grandmothers, HIV positive

## Abstract

**Background:**

Despite enormous interventions aimed at preventing mother-to-child transmission (MTCT) of HIV, cultural practices on mixed infant feeding remain prevalent. Complementary food provision to infants seems to be the most common and acceptable form of infant feeding highly endorsed by grandmothers.

**Aim:**

This study aimed to explore the perceptions of grandmothers and HIV-infected mothers on infant feeding practices in the context of prevention of mother-to-child transmission.

**Setting:**

This study was undertaken in two primary healthcare facilities in the Mpumalanga province, South Africa.

**Methods:**

A qualitative exploratory narrative design was used to conduct four focus group discussions with 32 purposefully selected grandmothers and 21 in-depth interviews with postnatal HIV-infected women in the two identified health facilities. Data were analysed using an interthematic inductive analysis approach, resulting in major themes and subthemes supported by participants’ excerpts.

**Results:**

Four themes emerged showing (1) high mixed feeding endorsement because of the need to adhere to conventional practices, strengthen the umbilicus and have fuller, fat and calmer babies; (2) infant feeding fallacies and stigma reflected by exclusive breastfeeding period confusion, breastfeeding scepticism and infant feeding stigma; (3) breastfeeding endorsement for other disease prevention and family support for breastfeeding and (4) conflict between traditional and western infant feeding approaches.

**Conclusion:**

Total elimination of MTCT of HIV in a rural context calls for targeted education for grandmothers addressing their perceptions and practices of infant feeding. The knowledge of the identified factors encouraging mixed infant feeding can assist in designing programmes to change community beliefs on infant feeding. Cultural, social and psychosocial factors should be addressed when making recommendations for exclusive breastfeeding for HIV-positive mothers.

## Introduction

Exclusive breastfeeding is an essential strategy for safeguarding childrens’ survival and good health, whilst also benefiting the mothers, irrespective of their HIV status (Mulol & Coutsoudis 2016; Oiye et al. 2017; WHO/UNICEF 2014). Exclusive breastfeeding for the first 6 months is the most appropriate infant feeding method for both HIV-positive and HIV-negative mothers (National Department of Health, Statistics South Africa, South African Medical Research Council, and ICF 2019; World Health Organization, United Nations Children’s Fund 2016). The benefits of exclusive breastfeeding for the first 6 months is that it lowers HIV transmission risk as compared to mixed infant feeding (World Health Organization, United Nations Children’s Fund 2016).

South Africa is amongst the 22 countries that have pledged to eliminate mother-to-child transmission (MTCT) of HIV by utilising the prevention of mother-to-child transmission (PMTCT) Option B+, which supports the provision of lifelong antiretroviral therapy to HIV-positive pregnant and lactating women without considering their CD4 count and infant feeding method of choice (Goga et al. 2018; WHO and United Nations Children’s Fund 2016). The estimated MTCT rate in East and Southern Africa is 8% (UNICEF 2017). In South Africa, the MTCT rate at6 weeks reduced from 3.6% in 2011 to 1.5% in 2016, with a post-6-week transmission rate of 3.1% (UNAIDS 2017; UNICEF 2017). Exclusive breastfeeding is, therefore, vital for encouraging total elimination of MTCT of HIV and for lowering the HIV transmission risk that might be caused by mixed infant feeding (World Health Organization, United Nations Children’s Fund 2016).

Whilst enormous strides have been made to reduce MTCT in South Africa by offering treatment provisions to mothers, exclusive breastfeeding is hard to practice, especially in a rural context. Recent global statistics reflect that only 41% of infants are breastfed exclusively in the first 6 months of life (UNICEF 2018). In South Africa, the rate of exclusive breastfeeding before 6 months is 32%, with 14% of infants drinking water and 18% eating other complementary foods whilst being breastfed (National Department of Health South Africa, Statistics South Africa, South African Medical Research Council & ICF 2017:29). Addressing the suboptimal infant feeding challenges, WHO (2014) has set global targets aimed at increasing exclusive breastfeeding rate by at least 50% by 2025. Exclusive breastfeeding for the first 6 months is the most appropriate infant feeding method irrespective of the mother’s HIV status (National Department of Health 2015:87; WHO and United Nations Children’s Fund 2016). HIV-positive women can breastfeed for at least 12 months and longer similar to the HIV-negative mothers. Infant feeding practices towards HIV exposed babies contribute to the burden of MTCT of HIV (South African National AIDS Council 2017:16).

Previous research has shown that postnatal MTCT is linked to practising mixed feeding rather than exclusive breastfeeding or exclusive formula feeding (Laher et al. 2012). Mixed infant feeding remains prevalent in some rural South African districts because of adherence to traditional practices or cultural factors (Bland et al. 2002; Mnyani et al. 2017). Therefore, feeding HIV-exposed infants continues to be challenging, especially in sub-Saharan Africa, where resources are limited (Yah & Tambo 2019) and cultural beliefs and practices prevail. National data on infant feeding indicate that breastfeeding is generally initiated immediately after birth; however, exclusive breastfeeding for the first 6 months does not usually occur regardless of the HIV status (Du Plessis et al. 2016; Mhlanga 2008; National Department of Health South Africa, Statistics South Africa, South African Medical Research Council & ICF 2017; West et al. 2019). Although women on the PMTCT programme attempt to breastfeed, exclusive breastfeeding seems insurmountable and difficult to sustain (Horwood et al. 2019). A recent study has shown that lack of knowledge contributes to poor infant feeding practices by HIV-infected mothers (Robb, Walsh & Nel 2018).

Numerous studies highlighted mixed feeding challenges, resulting in poor adherence to exclusive breastfeeding recommendations for both HIV-exposed but uninfected and HIV-unexposed children (Kinuthia et al. 2010; Ladzani et al. 2011; Morgan et al. 2010; Rossouw et al. 2016). Studies have shown that the act of mixed feeding generally occurs by providing breast milk together with water, tea and complementary food items (Swigart et al. 2017), breast milk with pure water (Ramara, Maputle & Lekhuleni 2010) and tea and water in addition to breast milk (Ferreira et al. 2018). Mixed feeding often starts as early as 1 month of age (Chaponda, Goon & Hoque 2017; Sibeko et al. 2009). Another recent study has noted 3–5 months as the start of mixed feeding (Du Plessis et al. 2016).

WHO (2014) indicates that societal beliefs aggravate the suboptimal rates of exclusive breastfeeding by caregivers, the lack of infant feeding support in both health facilities and communities and poor knowledge of the risks of mixed feeding. As caregivers, grandmothers play an influential role concerning the decisions taken on infant feeding (Chaponda et al. 2017). Their children often adhere to infant feeding suggestions they make out of respect for their parents (Chaponda et al. 2017). In a study conducted in the state of São Paulo, grandmothers were also found to be influential in giving complementary foods to infants before the age of 6 months. A review study showed that grandmothers take part in the upbringing of infants, as such, they are considered to be custodians of ‘traditional’ infant feeding knowledge (Kerr 2008; Negin et al. 2016). Grandmothers are also perceived to be ‘culturally designated advisors and caregivers’ (Aubel 2012) for both mother and child, even in the context of PMTCT. Several studies have shown that grandmothers’ own experiences of infant feeding influenced the feeding methods used for their grandchildren (Chaponda et al. 2017; Kafulafula et al. 2010; Negin et al. 2016).

### Problem statement

There are numerous interventions aimed at eliminating MTCT. However, adherence to traditional practices and cultural beliefs for mixed infant feeding remains rampant in some rural South African districts. HIV-infected mothers in a rural context continue to face myriad of challenges regarding infant feeding, whilst grandmothers remain custodians of ‘traditional’ infant feeding knowledge (Kerr 2008; Negin et al. 2016) even in the context of PMTCT. Risky behaviours, including mixed feeding habits, perpetuate HIV transmission amongst infants. There is a drive to encourage exclusive breastfeeding; however, provision of supplementary foods with breast milk needs further exploration (Sayed & Schönfeldt 2018). Early introduction of complementary food to HIV-exposed babies makes them more susceptible to sickness, such as diarrhoea, which might compromise their immune system. In a rural context, mixed feeding continues to be the most common and acceptable practice of infant feeding endorsed by grandmothers. Context plays a significant role in determining infant feeding approach (Chaponda et al. 2017). Infant feeding in the context of PMTCT is challenging, especially for HIV-infected mothers in rural areas. As outlined in the literature, grandmothers are central to infant feeding responsibilities, even in the context of HIV. It is, therefore, crucial to understand grandmothers and HIV-infected mothers’ perceptions of infant feeding to design context-specific interventions that would accelerate a total elimination of MTCT of HIV.

### Research purpose

This study explored grandmothers’ and HIV-infected mothers’ perceptions of infant feeding practices in the context of PMTCT.

## Research methods

### Study design and setting

A qualitative, exploratory, narrative design was used to explore grandmothers’ and HIV-infected mothers’ narratives of infant feeding practices in two primary healthcare (PHC) facilities of the Dr J.S. Moroka local municipality in the Mpumalanga province. This study is a subsection of the PhD study conducted in 2011 exploring the ‘intersecting narratives of PMTCT among HIV positive women, grandmothers and healthcare providers in a rural South African district’ (Mlambo 2014).

### Population and sampling

The study population comprised grandmothers and HIV-positive women utilising the identified two PHC facilities for routine health services and PMTCT services. In all, 32 grandmothers and 21 HIV-infected mothers were purposefully selected to explore their infant feeding practices in the context of PMTCT.

### Data collection

Thirty-two grandmothers participated in the four focus group discussions (FGDs) in the two identified PHC facilities. Grandmothers were randomly selected during their routine monthly chronic sickness check-up to participate in the FGDs, and none of them refused to participate in the study. A total of 6–10 grandmothers participated in each of the four FGDs, allowing them to share their wider expression of norms, values and opinions regarding infant feeding in the context of PMTCT. A trained HIV counsellor recruited them whilst waiting to be served, and FGDs took place after their consultations (Mlambo 2014). Grandmothers were selected based on the criterion that they had looked after a grandchild before, and to ensure confidentiality, there were no blood relations between grandmothers and HIV-infected women who participated in the study. Grandmothers gave informed consent prior to participation in the study. The FGDs were facilitated by the first author in a private space within the PHC facility, and they lasted for approximately 1–2 h (Mlambo 2014).

With regards to the HIV-infected women, the original study recruited 34 women receiving prenatal and postnatal services in the identified PHC facilities to participate in the study; however, only 29 purposefully selected women agreed. Of the five refusals, one had language challenges and the others had other domestic obligations to pursue (Mlambo 2014). This paper only focused on 21 in-depth interviews conducted with HIV-infected mothers receiving postnatal care services to explore their infant feeding practices in the context of PMTCT. The other eight in-depth interviews with HIV-positive women were excluded because they were still pregnant at the time of the study. The first author conducted in-depth interviews and they lasted for approximately 45 min per participant.

Both grandmothers and HIV-infected mothers responded to the following two general open-ended questions: ‘What are your perceptions of infant feeding in the context of HIV and PMTCT?’ and ‘What do women in your community say about infant feeding in the context of PMTCT?’ Probing questions included questions about current infant feeding practices and the age of complementary food introduction. Before responding to these questions, the researcher explained to the participants that early infants refer to babies aged 0–6 months. These infant feeding questions were based on the South African Department of Health PMTCT guidelines, advocating for exclusive breastfeeding for the first 6 months (South African National AIDS Council 2010). Both the FGDs and in-depth interviews were conducted using the indigenous Setswana language (Mlambo 2014).

### Data analysis

In preparation for data analysis, data were transcribed and translated from Setswana to the English language. Data were analysed using interthematic inductive analysis, which involved multiple readings of transcripts, generating codes and comparing emerging codes across different study participants. This led to a generation of major themes and subthemes (Creswell 2015). Disagreements resulting from the generated themes were discussed amongst the authors, and congruence was reached.

### Rigour of the study

The trustworthiness of research findings was ensured by adhering to the key principles, which included credibility, transferability, dependability and confirmability (Shenton 2004). These entailed utilising the triangulation principle, which permitted the use of multiple data collection sources. The participants’ characteristics, methodological descriptions and data analysis processes were also described. Findings were interpreted from the participant’s perspective.

## Findings

### Demographics

Table 1 shows that most of the grandmothers who participated in the FGDs were between the ages of 56–60 years (23%) and 71–75 years (23%). A high number of grandmothers were married (53%) and very few had no schooling (15.6%). Most grandmothers have 1–2 children (62.5%) with 5–7 grandchildren (32%).

**TABLE 1:** Grandmothers’ demographic characteristics.

Demographics	*n*	%
**Age (years)**		
40–45	2	6.7
46–50	3	10.0
51–55	6	20.0
56–60	7	23.3
61–65	3	10.0
66–70	2	6.7
71–75	7	23.3
76 and above	2	6.3
**Marital status**		
Married	17	53.1
Single	6	18.8
Widowed	7	21.9
Divorced/separated	2	6.3
**Education**		
No schooling	5	15.6
Primary	14	43.8
Secondary	13	40.6
**Employment status**		
Pensioner	16	50.0
Not pensioner	16	50.0
**Number of own children**		
1–2 children	20	62.5
3–4 children	11	34.4
5–6 children	1	3.1
**Number of grandchildren**		
1–2	9	29.0
3–4	8	25.8
5–7	10	32.3
8–11	4	12.9

### HIV-infected mothers’ demographics

In all, 21 HIV-infected mothers receiving postnatal services participated in in-depth interviews. Their ages ranged from 21 to 43 years and they were mostly single (18 women) with secondary education (15 women) (Mlambo 2014; Mlambo et al. 2018).

### Infant feeding perspectives amongst grandmothers and HIV-infected women

#### Thematic analysis findings

**FIGURE 1:**
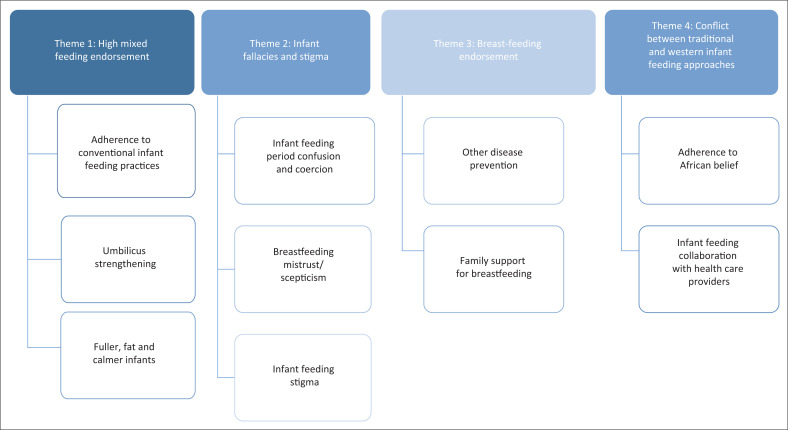
Themes of infant feeding perspectives amongst grandmothers and HIV-infected mothers.

The results reveal four themes highlighting grandmothers’ and HIV-infected mothers’ perceptions of infant feeding practices (Figure 1).

**Theme 1: High mixed feeding endorsement:** The findings show high mixed feeding endorsement amongst rural grandmothers and HIV-infected mothers because of a need to adhere to conventional infant feeding practices, strengthening umbilicus and having fuller, fat and calmer infants.

- **Adherence to conventional infant feeding practices**

Most grandmothers endorsed mixed feeding because of their belief that infants need to eat complementary foods. As reflected by the narration from one grandmother, most of them shared how mixed feeding is conducted in their communities.

‘Yes, an infant [*0–6 months*] should eat soft porridge! We raise them eating soft porridge [*maize*]! They eat soft porridge [*maize*]! We make it a bit softer, mixing it with milk. And it is not like we feed them soft porridge the whole day, they only eat it in the morning, during lunch and in the evening … An infant is meant to eat soft porridge [*maize*].’ (Grandmother 1, FGD 1)

To add on that, two other grandmothers said:

‘Breastfeeding goes hand in hand with complementary food …’ (Grandmother 3, FGD 4)

‘[*W*]hen you have an infant [*0–6 months*] at home, we give him/her soft porridge. After that, you make lukewarm water for him/her [*to drink*].’ (Grandmother 2, FGD 1)

Another grandmother added the period when mixed feeding conventionally occurs:

‘When the baby is two or three weeks old … you also boil water and give him/her with a bottle. That water is called “ntsu,” but it must be the water that has previously boiled …’ (Grandmother 3, FGD 1)

An HIV-infected mother explained how she feeds her infant:

‘I give her breast milk only. I have not started to give her water. Another thing that I give her is the medication for the fontanel [*anterior fontanel*] … When she is suffering from the “fontanel,” I take her to that person [*traditional healer*], then she drinks it … When she drinks it, she is fine. She does not become irritable. It is from traditional healers. They cook it and then we give her to drink …’ (Participant 12, 25 years, single)

Some HIV-infected mothers concurred with grandmothers’ perspectives highlighting the role grandmothers play in mixed infant feeding as highlighted below.

‘… [*T*]he grannies would say give him low water. They say you are supposed to make him drink low [*lukewarm*] water …’ (Participant 21, 43 years, single)

- **Umbilicus strengthening**

Mixed feeding was also believed to strengthen infants’ umbilicus:

‘Hey, I really do not understand why [*we should introduce complementary food only after six months*] because the baby has to eat to be strong. He/she has to eat! We are not refusing to follow the rules given at the clinic, but the baby has to eat! The inside of the navel is strengthened by food. The baby has to eat [*solids*], if not, the navel will start groaning, and that will make him/her cry … He/she has to get something that can relieve the tummy from groaning.’ (Grandmother 1, FGD 1)

In support of the above statement, another grandmother concurred by highlighting the impact of mixed infant feeding on the infants’ umbilicus:

‘… [*A*]nd even if you can just feed him three spoons [*of soft porridge*] it’s fine so that it can stop the umbilicus from groaning. If the umbilicus groans, he/she will start feeling cramps and discomfort; just like when it’s cloudy, the baby cries a lot.’ (Grandmother 1, FGD 2)

- **Fuller, fat and calmer babies**

The results further show that several grandmothers highlighted a need to stop the baby from crying as one of the reasons for persisting with mixed feeding:

‘We do not like the fact that the child is supposed to eat [*solids*] after six months. [*This is*] because the child will keep on crying due to not being full. When the child is three weeks old, he/she must start eating soft porridge so that he can stay fuller for longer …’ (Grandmother 2, FGD 1; Grandmother 7, FGD 4)

Some HIV-infected mothers also supported this point:

‘He eats soft porridge. I started giving him when he was three months old towards the end. It is because he was crying. You know when the baby is crying, he/she just continue crying if the bottle does not make him full.’ (Participant 1, 25 years, single)

Other grandmothers also continued to highlight the importance of having fuller babies:

‘[*I*]t’s a common practice [*in this community*] that the baby is given [*complementary*] food to grow up because these children … don’t get full from breastfeeding alone. So when you give him soft porridge, he/she gets full.’ (Grandmother 1, FGD 4; Grandmother 2, FGD 4)

Another grandmother indicated that mixed feeding is encouraged by baby food preference:

‘Babies are not alike,. some like being breastfed with [*complementary*] food and others don’t like being breastfed. When you give him some soft porridge, he will eat and even sleep …’ (Grandmother 4, FGD 4)

‘I was saying that even the breast milk is not the same, some are weak and some are strong and some babies don’t get full and you find the child crying non-stop and now after he has eaten [*it stops*] …’ (Grandmother 2, FGD 4; Grandmother 1, FGD 4)

**Theme 2: Infant feeding fallacies and stigma:** This study further found infant feeding misconceptions amongst rural grandmothers and HIV-infected mothers, which were largely because of infant feeding period confusion and coercion, breastfeeding mistrust/scepticism, and poor information on infant feeding guidelines and infant feeding stigma.

- **Infant feeding period confusion and coercion**

Their uncertainty regarding the breastfeeding period reflected infant feeding confusion amongst grandmothers:

‘The ladies in the olden days used to respect they were breastfeeding. Nowadays, when they [*young women*] deliver at the hospital, they say do not breastfeed the baby, we do not know what they see at the hospital. Others say you can breastfeed for a month … we do not know why doctors are saying that.’ (Grandmother 3, FGD 2)

The same confusion was reflected by other HIV-infected mothers, as indicated below:

‘Isn’t they say when you are HIV positive, you can give the baby breast milk up to certain months, I am not sure as to whether it is four months or how many months.’ (Participant 10, 30 years, single)

‘I feel very free to feed him/her … I have not started feeding him/her now; he/she drinks formula milk. I chose the bottle because I am not supposed to breastfeed. I don’t know why [*though*], I think it’s because they [*health providers*] assume that I will infect him/her. I once heard another lady … saying you can breastfeed up to six months, I am not sure if it’s true or whether you can breastfeed for a long period.’ (Participant 21, 43 years, single)

Some HIV-infected mother perceived breastfeeding as some form of coercion by healthcare providers:

‘Now you are forced to give the baby the breast. Even if you are sick, somehow you have to give the baby the breast …’ (Participant 4, 28 years, single)

- **Breastfeeding mistrust/scepticism**

Some HIV-infected mothers cited lack of trust as a reason to formula feed the baby. Two other HIV-infected mothers echoed their further scepticism in breastfeeding:

‘I was afraid that I would infect her. I had not started the treatment, so I thought I should give the baby a bottle. Even at home, they suggested that I give him the bottle … Giving the child a bottle is something we are used to, [*people*] will not ask what are you doing, what are you not doing in another way. It is more common among youth.’ (Participant 24, 21 years, single)

‘… [*T*]hat I am scared to give him the breast. That is why I started with formula … I am not able to understand the baby’s status when I am like this …’ (Participant 5, 33 years, single)

- **Infant feeding stigma**

HIV-infected mothers had conflicting views reflecting stigma challenges on formula feeding and breastfeeding. One mother who supports formula feeding said:

‘There is no person who takes the breast out to feed. People in this community give children formula milk. Don’t you know how the ladies are? I do not want my child to take the breast; I want my child to drink from the bottle; her father is working; I want her to drink formula milk.’ (Participant 10, 30 years, single)

Another mother who supports breastfeeding said:

‘Another problem is that people talk and you will find that they are not well-informed. When you start formula-feeding, they then say, she is sick. Meaning that as a woman, you are only supposed to breastfeed, because the minute you give formula milk, it means you are positive [*HIV+*], and it raises a whole lot of questions … Even if you can say you have rash [*on the nipple*], they do not believe you …’ (Participant 25, 27 years, single)

**Theme 3: Breastfeeding endorsement:** This study also reveals breastfeeding advocacy amongst grandmothers and HIV-infected mothers because of disease prevention and family support of breastfeeding.

- **Other disease prevention**

A few grandmothers appreciated breastfeeding as a suitable option to be used in the context of PMTCT:

‘But what remains is that the mother’s milk has protection against a lot of diseases … Even if the mother is HIV positive, she should breastfeed for 6 months and thereafter she can switch to formula.’ (Grandmother 2, FGD 3)

‘The baby must be breastfed because formula milk is expensive and the mother’s milk has a lot of vitamins, so she must breastfeed so that the baby can grow up.’ (Grandmother 1, FGD 4)

- **Family support for breastfeeding**

HIV-infected mothers indicated family support for breastfeeding:

‘My family told me that breast is healthy for my child because it will not make him/her sick … but the bottle is not good because it needs too much care. It needs too much care.’ (Participant 8, 33 years, single)

Another HIV-infected mother shared what the family highlights with regards to breastfeeding:

‘… The breast is easy and soft, and the dummy is hard … I once tried [*to give formula*], and the baby could not suck it, but when I give her the breast, she takes it well.’ (Participant 14, 23 years, single)

**Theme 4: Conflict between traditional and western infant feeding approaches:** The results further reveal the conflict between traditional and western infant feeding approaches reflected by a need to adhere to the African belief and encouraging transparency on infant feeding practices.

- **Adherence to African belief**

Grandmothers shared the approach that they use to harmonise their infant feeding practices:

‘It is our culture. If health providers say this [*regarding infant feeding*], when we come to the clinic we practise what they want [*do not mix feed*], but when we get home, we do what we [*normally*] do [*mixed feed*] because these kids are born with hunger. …!’ (Grandmother 4, FGD 1)

Similarly, another grandmother echoed the same words saying:

‘… Yes whilst at the clinic we listen to them when they speak, however, when we get home, I give the baby what I normally give him /her.’ (Grandmother 2, FGD 1)

Similarly, some HIV-infected mothers supported what grandmothers said:

‘They teach us about infant feeding, but when we get home, we do it our way because the child will be crying. Everyone who is here [*at the clinic*], no one can tell me that her child is not eating food. Because I am here, I will say I have never given my baby any complementary food, but when you get home, you give him/her …’ (Participant 12, 25 years, single)

- **Infant feeding collaboration with healthcare providers**

To address these conflicting infant feeding practices, grandmothers advocated for a collaborative relationship with healthcare providers to address infant feeding challenges. A few grandmothers reflected on this saying:

‘What I ask from the clinic staff and what we say as grandmothers is that let us work together. They should not reprimand us for what we do at home with these babies. Because we live with these children, we stay with them. We do bring them [*children*] at the stipulated times [*for immunisation*] that we are given. They should not blame us. Let us work together.’ (Grandmother 3, FGD 1)

‘Yes, yes, as to how they [*babies*] should eat yes, let us work together! We are also home nurses!’ (Grandmother 3, FGD 3)

### Ethical consideration

University of the Witwatersrand Human Research Ethics Committee (Medical), Ethical Clearance Number: M110666, 20/07/2011. The managers of the identified primary healthcare facilities also permitted the study. Both grandmothers and HIV-infected mothers gave informed consent before participation in the study, and no names were used during data collection to ensure confidentiality. Pseudonyms were used when conducting the focus group discussions.

## Discussion of findings

This study aimed to explore grandmothers’ and HIV-infected mothers’ infant feeding practices in the context of PMTCT. We found mutual agreement between grandmothers and HIV-infected mothers reflecting endorsement of mixed early breastfeeding and formula feeding because of the need to adhere to customary infant feeding methods, umbilicus strengthening and having fuller, fat and calmer babies. This mutual consensus on mixed feeding is primarily led by grandmothers who often instruct their children (HIV-infected mothers) to mixed feed as a way of adhering to conventional beliefs around infant feeding. Undoubtedly, this reveals that even in the context of PMTCT, respect for culture presenting itself in the form of grandmother’s advice on infant feeding matters prevails. Grandmothers are known to be the ‘custodians of infant feeding knowledge and culturally designated advisors’ for infant feeding (Aubel 2012; Kerr 2008; Negin et al. 2016). This suggests that exclusive breastfeeding in the context of PMTCT remains a challenging practice for HIV-infected mothers (Yah & Tambo 2019).

Unlike the study of Sayed and Schönfeldt (2018) that found high reliance on commercial cereals for mixed feeding, our study shows soft porridge [*maize*], lukewarm water and traditional herbs as the most common complementary foods given to the infants. Surprisingly in our study, tea is not mentioned, as was the case with other studies (Ferreira et al. 2018; Swigart et al. 2017). Our study also shows that, in this context, women strongly believe that provision of soft porridge to the infant strengthens the umbilicus. It could, therefore, be deduced that, in a rural context, culture is central to infant feeding issues as there are beliefs and community practices that paint a picture of an acceptable infant feeding form and its functions. Total elimination of mother-to-child transmission (EMTCT) of HIV can be realised if grandmothers’ stance on infant feeding matters is addressed holistically through partnership formation for better health system improvement.

Our study further reveals that mixed feeding typically starts when the infant is less than a month old, with only a few cases highlighting 3 months as an initial breastfeeding period. Similarly, other recent studies have shown that early introduction of supplementary food is still a common practice in South Africa (Nieuwoudt, Manderson & Norris 2018; Sayed & Schönfeldt 2018) and it starts within 1 month of baby’s birth (Chaponda et al. 2017; Sibeko et al. 2009) or from 3 months of age (Du Plessis et al. 2016). This reflects a need to change how infant feeding messages are communicated in the context of PMTCT. The policies set on infant feeding are challenging to implement in a rural context. HIV-infected mother and grandmother dyads are crucial in planning for infant feeding.

Again, due to cultural and societal beliefs, this study found that grandmothers mostly perceived breast milk to be of different quality for each mother, suggesting that some breast milk keeps the baby fuller for a longer time compared to others. One reason for believing that the milk is not satisfying is if the infant is not latched correctly. This suggests a need for breast-latching education to mothers who have just given birth. Grandmothers can play a pivotal role in showing new mothers how to latch their infants in a rural context.

As highlighted under Theme 2, this study found infant feeding fallacies about the ideal exclusive breastfeeding period. Understanding facts about the exclusive breastfeeding period is crucial for a total EMTCT of HIV. This suggests a need to strengthen education on the importance of exclusive breastfeeding, emphasising the ideal periods. This study also found that some HIV-infected women mistrusted or doubted the messages on exclusive breastfeeding because of their HIV status. Similarly, West et al. (2019) found that despite infant feeding counselling, some HIV-positive mothers feared vertical HIV transmission. There is a need to strengthen educational messages in any given context.

On the other hand, HIV-infected mothers in this study felt stigmatised on whichever infant feeding method they chose. Both infant feeding methods seem to raise questions from family and peers regarding their HIV status, especially if it is perceived to be uncommon. Similarly, Nabwera et al. (2017) found that since exclusive breastfeeding was not a norm, it often raised questions from fellow community members, including family. A study conducted in Soweto also found that exclusive breastfeeding was more linked to one being HIV positive, instead of considering it as a strategy for infant health promotion (Nieuwoudt & Manderson 2018). This suggests that although the importance of exclusive breastfeeding is acknowledged, the culture and the ability to make decisions about infant feeding is a challenge in the context of PMTCT. Grandmothers should be capacitated with the right knowledge as they are regarded as the custodians of knowledge (Kerr 2008; Negin et al. 2016). In contrast, a study conducted in Kwa-Zulu Natal in South Africa found that women practised exclusive breastfeeding based on the advice received from the healthcare providers (Horwood et al. 2019). This suggests the need for the involvement of healthcare providers, HIV-infected mothers and grandmothers in infant feeding planning in a rural context.

This study found that breastfeeding was advocated for preventing diseases and was practised based on family recommendation; however, it was not necessarily exclusive breastfeeding. It is therefore important to acknowledge grandmothers’ attitude towards infant feeding to ensure its integration into healthcare delivery (Kerr et al. 2008). Poor knowledge of infant feeding guidelines in this study pose conflict between traditional and western infant feeding systems. Our study reveals that grandmothers see themselves as nurses at home who can implement traditional infant feeding approaches. Their role as culturally designated advisors and caregivers (Aubel 2012) endorse this claim. Their call for collaboration on infant feeding matters points to their readiness in working with healthcare providers.

## Recommendations

Targeted education for grandmothers addressing their perceptions and practices on infant feeding is crucial. The knowledge of the factors encouraging mixed infant feeding should be utilised in designing programmes to change community beliefs on infant feeding. Programmes promoting exclusive breastfeeding for HIV-positive mothers should be sensitive to socio-cultural expectations and can utilise grandmother’s influence on breastfeeding. In a rural context, partnership formation with grandmothers can greatly improve service provision for HIV-positive women, including preventing MTCT of HIV. PMTCT guidelines should address this and reveal the importance of partnership with grandmothers to facilitate total EMTCT of HIV.

## Conclusion

For MTCT of HIV to be eliminated in a rural context, there should be targeted education for grandmothers on infant feeding in the context of PMTCT. Grandmothers should be used as educators for infant feeding. Cultural, social and psychosocial factors should be addressed when making recommendations for exclusive breastfeeding for HIV-positive mothers. Healthcare providers remain crucial in delivering the right messages to a family unit regarding the importance of exclusive breastfeeding. These could be improved by conducting health system, community and policy level interventions.
